# The profiles of angiology and vascular surgery academic leagues and their effectiveness in specialty education

**DOI:** 10.1590/1677-5449.006318

**Published:** 2019-02-18

**Authors:** Stephani Andreoni, Denise Colosso Rangel, Giselle Cristina Bitencourt Garcia de Sá Barreto, Rafael Henrique Isaias Rodrigues, Henrique Mozart Toso Alves, Lucas Azevedo Portela

**Affiliations:** 1 Universidade de Mogi das Cruzes (UMC), Faculdade de Medicina, Mogi das Cruzes, SP, Brasil.

**Keywords:** medical education, student, academic performance

## Abstract

**Background:**

Academic leagues are extracurricular student organizations that are supervised by professors on the faculty of a higher education institution and are dedicated to improving knowledge in certain areas. There has recently been a marked growth in the number of new leagues, giving undergraduate students access to additional information, lectures, internships, and conferences. Angiology and vascular surgery is one of the specialties that have such organizations.

**Objectives:**

To determine the academic profile of the angiology and vascular surgery academic leagues at medical schools in the state of São Paulo, Brazil, and investigate the academic performance of their members.

**Methods:**

This is a cross-sectional, descriptive, and analytical study that recruited undergraduate medical students who had joined angiology and vascular surgery academic leagues in the state of São Paulo. On-line questionnaires were used to collect data on each league and the students took examinations to determine their performance.

**Results:**

Most leagues had an entrance exam for enrollment, with an obligatory introductory course. Monthly theoretical lectures were held by 42.9% of the leagues. Practical activities were provided by 85.7% of the leagues. The majority of the leagues (71.4%) were involved in scientific research. Paired-sample comparison of students’ performance in the exams revealed a significant increase in mean scores, from 61.1 to 72.6 (p < 0.05).

**Conclusions:**

The angiology and vascular surgery academic leagues in the state of São Paulo all function in a similar manner, but the range of theoretical, practical and scientific activities they offer are not uniform. For the sample investigated, the academic leagues appear to be effective at teaching angiology and vascular surgery during undergraduate courses.

## INTRODUCTION

 Medical science teaching has a history going back millennia and as developments emerge over time new discoveries and technologies have influenced medical education. Since the very earliest days of medical training, it was felt that there was a need for a consultant or tutor or even a group of people who shared the same interest in a research problem. Technical development, social responsibility, and humanitarian behavior were essential elements of medical education to train professionals capable of identifying and treating health problems in other human beings. 1

 In the mid 1920s, groups with objectives and interests in common began to grow and exchange information, experiences, and activities (with a tutor as facilitator), enabling undergraduate students to develop their skills. The seed from which Brazil’s academic leagues would grow had been sown. 2

 The academic leagues are student organizations supervised by a professor from a teaching institution that work to improve knowledge in specific areas through extracurricular activities. Factors that possibly motivate undergraduate students studying Medicine to participate in the academic leagues include: contact with medical practice, acquisition of experience, interaction with colleagues, identification with a group, professional qualification, and a need to overcome possible curricular deficiencies. 3

 The first league established in Brazil was the League to Fight Syphilis (Liga de Combate à Sífilis), in 1920, at the Universidade de São Paulo Medical Faculty. 3 During the years under dictatorship, questions were asked about the essence of the teaching provided at universities. 4 During the 1990s, many leagues were founded all over Brazil. This occurred against a background of sweeping curricular reforms and intense political and academic debate about professional medical training. 2

 In general, the academic leagues offer undergraduates access to theoretical lectures, courses, symposia, scientific research, conferences and, in some cases, supervised medical care activities. 3 ^,^ 4

 Over the years, associations of academic leagues have been formed, such as, for example, the Brazilian Society of Cardiology Leagues (Sociedade Brasileira de Ligas de Cardiologia), which has almost 80 member leagues and organizes symposia and conferences, illustrating the organizational level that such entities can attain. 5 The São Paulo Academic League for Angiology and Vascular Surgery (Liga Acadêmica Paulista de Angiologia e Cirurgia Vascular), an adjunct of the Brazilian Society of Angiology and Vascular Surgery, was founded in 2013 and already has approximately 10 associate leagues in São Paulo state. 

 The objective of this study was to trace the profiles of angiology and vascular surgery academic leagues at medical schools in the state of São Paulo, Brazil. 

## METHODS

 This is a cross-sectional, descriptive, analytical study conducted with 56 medical students who were members of angiology and vascular surgery academic leagues at medical schools in the state of São Paulo. This study was approved by the Research Ethics Committee at the Universidade de Mogi das Cruzes, under hearing number 2.216.240 (ethics approval certificate number 69718117.6.0000.5497) on August 13, 2017. 

 An initial survey was conducted of medical schools in the state of São Paulo that have angiology and vascular surgery academic leagues. All of the students and presidents involved agreed to participate voluntarily and signed free and informed consent forms. 

 Teaching effectiveness was determined by application of two identical assessment exams, containing 20 multiple choice questions, randomized across the students, before (test I) and after (test II) the leagues’ 2017 academic year. These assessment tests covered the material that comprises the basic knowledge of the angiology and vascular surgery specialty (anatomy, propadeutics [workup], natural history, and etiology and pathophysiology), taken from two Brazilian references on the specialty (text books). 6 ^,^ 7 Both exams contained the same questions, but the sequence was changed for test II. The method used to develop these test papers has been described elsewhere. 8 ^-^ 10 The tests were administered to students who took part in the leagues, using the ProProfs® 11 on-line platform). Students were not given the test answers. Fifty-six student volunteers took part in the assessments. The analysis based on comparison of mean test scores was conducted with a 0.05 (5%) statistical error and 95% confidence intervals. The sample comprised more than 30 subjects, which ensured a normal distribution and homogeneity of variance (constant variance). Mean test I score was compared to mean test II score using Student’s *t* test. 

 The profiles of the academic leagues were evaluated using an on-line questionnaire that was completed by the president of each league on the Google Forms® 12 platform). The objective was to investigate how these entities function within medical schools and what is offered to students who sign up, in terms of internships, lectures, scientific output, and extracurricular activities. 

## RESULTS

 The survey of medical schools in the state of São Paulo identified a total of 59. Just 10 of these have an angiology and vascular surgery league. Seven leagues agreed to participate voluntarily in the study: six in the greater São Paulo area (86%) and one in upstate São Paulo (14%). These leagues were founded between 2008 and 2017. The number of new members they enrolled in 2017 varied from six to 10 students. 

 The leagues have administrations organized as president, vice-president, treasurer, etc. The administrations of the leagues investigated have terms varying from 1 year to 1 and a half year. The oldest of these leagues, founded in 2008, has had six terms to date. 

**Figure 1:**
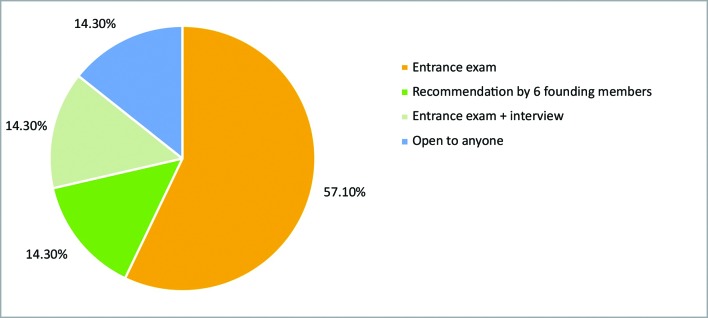
Criteria for enrollment in leagues.

 Enrollment of new members involves a written test of theory in 57.10% of the leagues. Figure 1 illustrates the distribution of enrollment systems. In 42.9% of the leagues, enrollment is limited by the number of places available (decided by the administration); in 28.6% enrollment is restricted by course year; and the remainder of the leagues do not set numerical limits on the number of students enrolled. 

**Figure 2:**
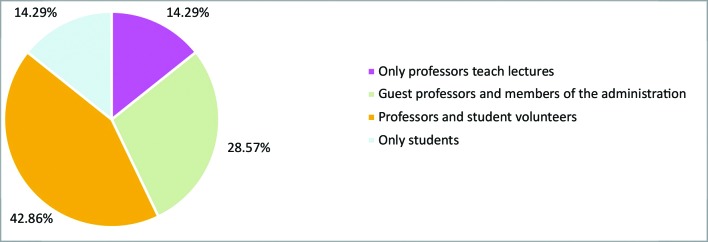
Distribution of lecturers.

 All of the leagues organized obligatory introductory courses that were a prerequisite for enrollment, with the primary objectives of presenting the material that would be tested in enrollment exams and to ensure a standard minimum level of knowledge for new members. With relation to lectures, 42.9% of the student organizations offered monthly lectures, 42.9% offered fortnightly lectures, and one of them held lectures weekly. A majority of the leagues (85.7%) ran a proportion of these lectures in partnership with other specialty leagues. Figure 2 illustrates the distribution of the lecturers. 

**Figure 3:**
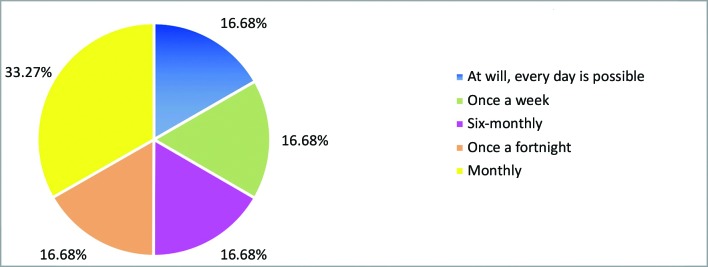
Frequency of practical internships.

**Figure 4:**
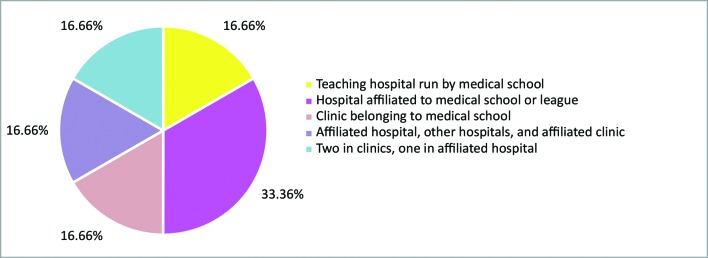
Distribution of practical internships.

 In addition to theoretical activities, 85.7% of the leagues ran supervised internships as practical activities. The frequencies of practical internships are illustrated in Figure 3 . Placements were clinical and/or surgical and were available to all members. The settings in which internships were offered are illustrated in Figure 4 . 

**Figure 5:**
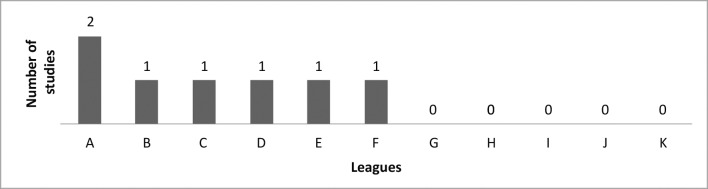
Number of scientific studies presented at conferences.

 Scientific activities (academic research) were part of the majority of the leagues’ programs (71.4%). Six out of seven of the leagues provide incentives for scientific activity, while 100% of the leagues’ students participated. The number of presentations at academic conferences per league is illustrated in Figure 5 . 

 A total of 56 students took the exams to test the leagues’ teaching effectiveness (tests I and II). A total of 34 students took test I and 34 students took test II. A total of 12 students took both tests. 

**Figure 6:**
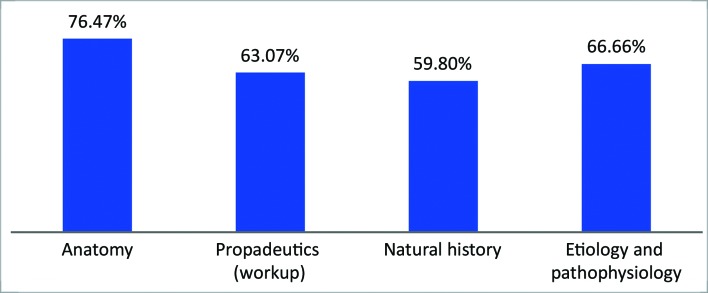
Percentages of correct answers by subject axis – Test I.

 In test I, the highest percentage of correct answers were for questions on vascular anatomy (76.4%). The lowest percentage was for the group of questions on natural history of the disease (59.8%), as illustrated in Figure 6 . 

**Figure 7:**
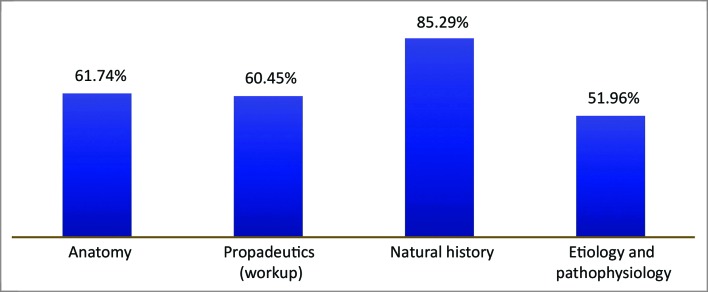
Percentages of correct answers by subject axis – Test II.

 In test II, the highest proportion of correct answers was for questions on natural history (85.2%), and the lowest was on the etiology and pathophysiology of vascular diseases (51.9%), as illustrated in Figure 7 . Analysis of the results obtained by paired sample comparison (students who took both test I and test II) revealed a statistically significant (p < 0.05) increase in mean test scores (from 61.1 to 72.6). Tests were scored from 0 to 100. The non-paired analysis did not detect the same difference. 

## DISCUSSION

 The basic objective of academic leagues is to help future doctors to accumulate knowledge; but, to date, they have been investigated little. 13 This study found that although there is a large number of medical schools in the state of São Paulo, very few of these institutions have an academic league focused on the angiology and vascular surgery specialty. The leagues that do exist, in turn, were founded recently, the oldest in 2008. 

 Practical activities can introduce students to the day-to-day reality of practicing the specialty, improving the patient-physician relationship, in addition to consolidating all of the knowledge taught within the university curriculum, by means of the introductory course and the lectures provided. This study found that the majority of leagues offer these activities, which is compatible with the literature. 14 ^,^ 15 Other studies suggest that these activities should be carried out in small groups, so that each student can spend more time with patients and teacher-student interaction is facilitated. 13 ^,^ 16

 The objective of the processes for selection of new students is to screen for those with greatest interest in the specialty. 8 This study observed a variety of criteria for enrollment in the leagues, but exams were the methodology most widely used, as reported elsewhere. 13 ^-^ 15 ^,^ 17

 It was also observed that the introductory course, lectures, and other face-to-face theoretical activities are offered by all of the leagues, which is similar to data available in the literature. 13 Characteristics such as the frequency of meetings, lectures in partnership with other organizations, lecturers, and restrictions on membership exhibited great variability across the leagues, as has been described previously. 15

 With regard to scientific activities, all of the leagues stated that they take part in conferences, present and publish scientific research, which is stimulated by the institutions to which they are affiliated. Many studies have shown the positive impact of these activities, demonstrating that the students’ primary objective is to build an outstanding CV. 14 ^,^ 18

 The students performed well in both of the exams set (scores exceeding 60%). The paired analysis of what was learned over the course of an academic year revealed a statistically significant increase in overall mean scores, demonstrating that performance had improved. No other studies in the literature have assessed the knowledge of students who are members of academic leagues. Previous studies have reported that the knowledge, experience, and development of scientific reasoning acquired through academic league activities are useful in many medical specialties and in correlated areas, 16 ^,^ 17 ^,^ 19 even when the student does not ultimately opt to practice the specialty in question. 13 ^,^ 20

## CONCLUSIONS

 The angiology and vascular surgery academic leagues in the state of São Paulo (Brazil) all function in a similar manner, but offer a varied range of theoretical, practical, and scientific activities. In the sample investigated, the academic leagues were effective for teaching angiology and vascular surgery to undergraduate students. 
