# Supplementary material for: The profiles of angiology and vascular surgery academic leagues and their effectiveness in specialty education
Source: J Vasc Bras. 2019 Feb 18;18:e20180063. doi: 10.1590/1677-5449.006318 (PMC6579523; doi:10.1590/1677-5449.006318)
Supplement: Translation

# Translation: O perfil das ligas acadêmicas de angiologia e cirurgia vascular e sua eficácia no ensino da especialidade

**Authors:** Stephani Andreoni, Denise Colosso Rangel, Giselle Cristina Bitencourt Garcia de Sá Barreto, Rafael Henrique Isaias Rodrigues, Henrique Mozart Toso Alves, Lucas Azevedo Portela

**Affiliations:** 1 Universidade de Mogi das Cruzes (UMC), Faculdade de Medicina, Mogi das Cruzes, SP, Brasil.

**Keywords:** educação médica, estudante, desempenho acadêmico

## Abstract

**Contexto:**

As ligas acadêmicas são organizações estudantis extracurriculares que contam com a supervisão de um docente vinculado à instituição de ensino e visam aprimorar o conhecimento em determinadas áreas. Verificou-se, nos últimos anos, um crescimento do número de novas ligas, nas quais os graduandos têm acesso a informações complementares através de aulas, estágios e congressos. A especialidade de angiologia e cirurgia vascular encontra-se entre aquelas que dispõem dessas organizações.

**Objetivos:**

Determinar o perfil acadêmico das ligas de angiologia e cirurgia vascular das faculdades de Medicina do estado de São Paulo e verificar o desempenho dos membros dessas ligas no ensino.

**Métodos:**

Este estudo transversal, descritivo e analítico incluiu graduandos de Medicina membros de ligas acadêmicas de angiologia e cirurgia vascular do estado de São Paulo. Foram utilizados questionários *on-line* para determinação dos dados de cada liga e foram aplicadas provas para verificação do desempenho.

**Resultados:**

O ingresso ocorreu prioritariamente por prova, com curso introdutório obrigatório. Realizaram-se aulas teóricas mensalmente em 42,9% das ligas. Quanto a atividades práticas, 85,7% das ligas as realizaram. A maioria das ligas (71,4%) relataram realizar atividades científicas. A comparação pareada do desempenho dos alunos nas provas demonstrou um aumento significativo na média, de 61,1 para 72,6 (p < 0,05).

**Conclusões:**

As ligas acadêmicas de angiologia e cirurgia vascular do estado de São Paulo apresentam modos de funcionamento semelhantes e um perfil heterogêneo quanto a atividades teóricas, práticas e científicas. A partir da presente amostra, as ligas acadêmicas aparentam ser efetivas no ensino de angiologia e cirurgia vascular durante a graduação.

## INTRODUÇÃO

 O ensino e a aprendizagem da ciência médica datam de milênios, e a evolução dos tempos, novas tecnologias e descobertas influenciaram esse processo. Desde os primórdios do ensino médico, sentiu-se a necessidade de haver um consultor/tutor ou até mesmo um grupo de pessoas que compartilhasse um mesmo interesse sobre um problema de pesquisa. O desenvolvimento técnico, a responsabilidade social e o comportamento humanitário deveriam fazer parte da educação médica para a formação de profissionais capazes de identificar e tratar problemas de saúde em outros seres humanos 1 . 

 Em meados da década de 1920, grupos com objetivos e interesses comuns começaram a crescer a partir de trocas de informações, experiências e atividades (com o auxílio de um tutor), possibilitando o desenvolvimento de habilidades durante a graduação. Assim, lançou-se a semente para o surgimento das ligas acadêmicas no Brasil 2 . 

 As ligas acadêmicas são organizações estudantis que contam com a supervisão de um docente vinculado à instituição de ensino e visam aprimorar o conhecimento em determinadas áreas, sendo esta uma atividade extracurricular. Entre os fatores motivacionais que levam os graduandos de Medicina a participarem das ligas acadêmicas podem estar: aproximação da prática médica, aquisição de experiência, interação com colegas, identificação com um grupo, qualificação profissional e tentativa de suprir possíveis insuficiências curriculares 3 . 

 A primeira liga criada no Brasil foi a Liga de Combate à Sífilis, em 1920, na Faculdade de Medicina da Universidade de São Paulo 3 . O período da ditadura forneceu um contexto que favoreceu o despertar de questionamentos acerca da essência dos ensinamentos oferecidos pelas universidades 4 . Já na década de 1990, muitas ligas foram criadas em todo o país. Esse fato coincidiu com um período repleto de reformas curriculares e intenso debate político e acadêmico sobre a formação profissional médica 2 . 

 Nas ligas acadêmicas, os graduandos têm, usualmente, acesso a aulas teóricas, cursos, simpósios, pesquisa científica, congressos e, eventualmente, atividades de assistência médica supervisionada 3 ^,^ 4 . 

 Ao longo dos anos foram surgindo também associações de ligas acadêmicas como a Sociedade Brasileira de Ligas de Cardiologia, que inclui quase 80 ligas e organiza simpósios e conferências, demonstrando o nível organizacional que essas entidades podem atingir 5 . A Liga Acadêmica Paulista de Angiologia e Cirurgia Vascular, adjunta à Sociedade Brasileira de Angiologia e Cirurgia Vascular, foi criada no ano de 2013 e já inclui aproximadamente 10 ligas associadas em todo o estado. 

 Este estudo tem como objetivo verificar o perfil e o ensino das ligas acadêmicas de angiologia e cirurgia vascular das faculdades de Medicina do estado de São Paulo. 

## MÉTODOS

 Trata-se de um estudo transversal, descritivo e analítico com 56 alunos de Medicina membros de ligas de angiologia e cirurgia vascular das faculdades de Medicina do estado de São Paulo. Esta pesquisa foi aprovada pelo Comitê de Ética em Pesquisa da Universidade de Mogi das Cruzes com parecer nº 2.216.240 (CAAE nº 69718117.6.0000.5497) em 13 de agosto de 2017. 

 Foi feito um levantamento inicial das escolas médicas do estado de São Paulo que contam com ligas acadêmicas de angiologia e cirurgia vascular. Todos os alunos e presidentes envolvidos aceitaram participar voluntariamente da pesquisa, assinando o termo de consentimento livre e esclarecido. 

 A determinação da efetividade no ensino foi realizada através de duas avaliações idênticas, com 20 questões de múltipla escolha, aleatorizadas entre os alunos, aplicadas antes (prova I) e após (prova II) o ano letivo das ligas em 2017. Essas avaliações contemplaram conteúdo de conhecimento básico da especialidade de angiologia e cirurgia vascular (anatomia, propedêutica, história natural e etiofisiopatologia) com duas fontes (livros-texto) de referência nacional da especialidade 6 ^,^ 7 . As provas continham as mesmas questões, em ordens modificadas para a prova II. O método de desenvolvimento desses questionários já foi previamente descrito 8 ^-^ 10 . As provas foram submetidas aos alunos participantes das ligas, com aplicação *on-line* pela plataforma ProProfs® 11 ). Os alunos não receberam o gabarito das provas. Cinquenta e seis alunos participaram voluntariamente das avaliações. Para a análise da comparação das médias em ambas as provas, foi definido um erro estatístico de 0,05 (5%), e todos os intervalos de confiança foram construídos com 95% de confiabilidade estatística. A amostragem foi superior a 30 sujeitos, o que garantiu uma distribuição normal e homocedasticidade (variância constante). Para comparação da média da pontuação da prova I com a da prova II, foi utilizado o teste T de Student. 

 A avaliação do perfil das ligas acadêmicas foi feita através de questionário *on-line* preenchido pelos presidentes das ligas pela plataforma Google Forms® 12 . O objetivo era determinar o funcionamento dessas entidades nas escolas médicas e o que é ofertado aos alunos participantes em termos de estágios, aulas, produção científica e atividades extracurriculares. 

## RESULTADOS

 O levantamento das faculdades de Medicina no estado de São Paulo resultou em um total de 59 escolas. Dessas, apenas 10 contavam com uma liga de angiologia e cirurgia vascular. Sete ligas participaram voluntariamente do estudo: seis da região da Grande São Paulo (86%) e uma do interior do estado (14%). A data de fundação dessas ligas variou entre 2008 e 2017. O número de novos membros ingressantes no ano de 2017 variou entre seis e 10 alunos. 

 As ligas dispunham de gestões, organizadas em presidente, vice-presidente, tesoureiro, etc. Entre as ligas pesquisadas, as gestões variaram entre um ano e um ano e meio de duração. A liga mais antiga, criada em 2008, já apresentou seis gestões até o momento. 

 O ingresso de novos membros ocorreu através de teste teórico escrito em 57,10% das ligas. A Figura 1 demonstra a distribuição das formas de ingresso. Quanto à restrição de ingresso, verificou-se que, em 42,9% das ligas, esta ocorre por limite de vagas disponíveis (determinada pela gestão); em 28,6%, por ano de graduação; e as demais não fazem restrições numéricas de alunos ingressantes. 

**Figura 1:** Formas de ingresso nas ligas.

 Como pré-requisito ao ingresso nas ligas, 100% delas realizaram um curso introdutório, com as principais finalidades de apresentar os conteúdos para a prova de ingresso e nivelar o conhecimento dos novos membros. Entre as entidades estudantis, 42,9% relataram oferecer aulas para seus membros mensalmente, 42,9% quinzenalmente, e uma delas semanalmente. A maioria das ligas (85,7%) ministraram parte dessas aulas em parceria com ligas de outras especialidades. A Figura 2 ilustra a distribuição dos ministrantes das aulas. 

**Figura 2:** Distribuição dos ministrantes de aulas.

 Além das atividades teóricas, 85,7% das ligas realizaram estágios supervisionados como atividade prática. A frequência de realização dos estágios está demonstrada na Figura 3 . Os estágios eram de cunho clínico-cirúrgico e disponíveis para todos os membros. O local de realização desses estágios é demonstrado na Figura 4 . 

**Figura 3:** Frequência da realização dos estágios práticos.

**Figura 4:** Distribuição dos estágios práticos.

 A atividade científica era uma realidade na maioria das ligas (71,4%). Houve incentivo dessa atividade em seis das sete ligas pesquisadas. A participação dos alunos ocorreu em 100% das ligas. A quantidade de trabalhos apresentados em congressos pelas ligas é demonstrada na Figura 5 . 

**Figura 5:** Quantidade de trabalhos científicos apresentados em congressos.

 Um total de 56 alunos responderam as avaliações para verificação da efetividade do ensino nas ligas (provas I e II). Responderam a prova I um total de 34 alunos, e outros 34 responderam a prova II. Um total de 12 alunos responderam ambas as provas. 

 Na prova I, houve um maior percentual de acertos de questões que envolveram o assunto anatomia vascular (76,4%). O menor percentual foi verificado no grupo de questões referentes ao assunto história natural da doença (59,8%), conforme mostra a Figura 6 . 

**Figura 6:** Índice de acertos por eixo de conhecimento da prova I.

 Na prova II, o maior índice de acertos foi sobre história natural (85,2%), e o menor sobre etiofisiopatologia das doenças vasculares (51,9%), conforme demonstrado na Figura 7 . Na análise dos resultados obtidos pela comparação de forma pareada (alunos que participaram tanto da prova I quanto da prova II), houve um aumento na média (de 61,1 para 72,6) das pontuações nas provas, com significância estatística (p < 0,05). As provas eram pontuadas de 0 a 100. Essa diferença não foi encontrada na análise não pareada. 

**Figura 7:** Índice de acertos por eixo de conhecimento da prova II.

## DISCUSSÃO

 As ligas acadêmicas possuem o objetivo básico de auxiliar na construção do conhecimento dos futuros médicos; porém, ainda hoje, têm sido pouco exploradas 13 . No presente estudo, constatou-se que apesar do grande número de faculdades no estado de São Paulo, poucas são as instituições que possuem uma liga acadêmica dedicada à especialidade de angiologia e cirurgia vascular. Essas ligas, por sua vez, têm pouco tempo de fundação, sendo a mais antiga inaugurada em 2008. 

 Atividades práticas podem aproximar o aluno da realidade diária da especialidade, melhorando o relacionamento com o paciente, além de consolidar todo o conhecimento transmitido pelo currículo da faculdade, pelo curso introdutório e pelas aulas ministradas. Na presente pesquisa, foi evidenciado que a maioria das ligas dispõe dessas atividades, o que é compatível com a literatura 14 ^,^ 15 . Outros estudos sugerem que tais atividades sejam feitas em pequenos grupos, para que o aluno possa ter mais tempo próximo ao paciente e a interação entre professor e aluno seja favorecida 13 ^,^ 16 . 

 O objetivo do processo seletivo dos novos membros é triar alunos com maior interesse pela especialidade 8 . No atual estudo, foram encontrados diversos critérios de ingresso, mas a aplicação de provas foi a metodologia mais utilizada, compatível com relatos anteriores 13 ^-^ 15 ^,^ 17 . 

 Verificou-se também que o curso introdutório, assim como aulas e outras atividades teóricas presenciais, estão presentes em todas as ligas avaliadas, dado semelhante ao encontrado na literatura 13 . Nos quesitos periodicidade de encontros, aulas em parceria, palestrantes e limite dos integrantes das ligas, notou-se grande variabilidade entre as ligas pesquisadas, como descrito em estudo prévio 15 . 

 Quanto ao desenvolvimento de atividades científicas, todas as ligas relataram participar de congressos e desenvolver trabalhos científicos, sendo estes estimulados pelas instituições as quais estão vinculadas. Diversos estudos reafirmam o aspecto positivo dessas atividades, demonstrando que o objetivo principal do aluno é a construção de um currículo diferenciado 14 ^,^ 18 . 

 Em ambas as avaliações realizadas, os alunos apresentaram bom desempenho (notas superiores a 60%). Na avaliação do aprendizado durante o período letivo, comparando-se dados pareados, constatou-se um aumento estatisticamente significante da média geral, determinando melhora comprovada no desempenho. Não há outros estudos na literatura que realizaram avaliações de conhecimento de alunos de ligas acadêmicas. Estudos prévios já relatavam que o conhecimento, a experiência e o desenvolvimento de raciocínio científico adquiridos nas atividades em uma liga acadêmica são úteis em diversas especialidades da medicina e áreas correlatas 16 ^,^ 17 ^,^ 19 , mesmo quando o aluno não opte por desempenhar posteriormente a especialidade em questão 13 ^,^ 20 . 

## CONCLUSÃO

 As ligas acadêmicas de angiologia e cirurgia vascular do estado de São Paulo apresentam modos de funcionamento semelhantes e um perfil heterogêneo quanto a atividades teóricas, práticas e científicas. A partir da presente amostra, as ligas acadêmicas aparentam ser efetivas no ensino de angiologia e cirurgia vascular durante a graduação.
